# Health-related quality of life in boys with constitutional delay of growth and puberty

**DOI:** 10.3389/fendo.2022.1028828

**Published:** 2022-11-25

**Authors:** Laura Kariola, Tero Varimo, Hanna Huopio, Sirpa Tenhola, Raimo Voutilainen, Silja Kosola, Jorma Toppari, Harri Sintonen, Päivi J. Miettinen, Taneli Raivio, Matti Hero

**Affiliations:** ^1^ Helsinki University Hospital, New Children’s Hospital, Pediatric Research Center, Helsinki University, Helsinki, Finland; ^2^ Kuopio University Hospital, Kuopio, Finland; ^3^ Department of Pediatrics, Kymenlaakso Central Hospital, Kotka, Finland; ^4^ Institute of Biomedicine, Research Centre for Integrative Physiology and Pharmacology, and Centre for Population Health Research, University of Turku, and Department of Pediatrics, Turku University Hospital, Turku, Finland; ^5^ Department of Public Health, University of Helsinki, Helsinki, Finland; ^6^ Translational Stem Cell Biology and Metabolism Research Program, Faculty of Medicine, University of Helsinki, Helsinki, Finland

**Keywords:** delayed puberty, constitutional delay of growth and puberty, letrozole, testosterone, health-related quality of life, 16D

## Abstract

**Introduction:**

Constitutional delay of growth and puberty (CDGP) is the most common reason for delayed puberty in healthy male adolescents. The main indication for medical treatment for this condition is psychosocial burden. However, to the best of our knowledge, no previous study has addressed the impact of puberty-promoting treatment on health-related quality of life (HRQoL) among boys with CDGP.

**Methods:**

We investigated HRQoL in 22 boys with CDGP, who participated in a randomized controlled trial in four Finnish pediatric endocrinology outpatient clinics between 2013 and 2017. The boys were randomized to receive either aromatase inhibitor letrozole (2.5mg/day; n=11) or intramuscular testosterone (1mg/kg/every 4 weeks; n=11) for 6 months and followed up to 12 months. HRQoL was assessed with a generic self-assessment 16D^©^ instrument developed and validated for adolescents aged 12 to 15 years. The 16D includes 16 dimensions (vitality, sight, breathing, distress, hearing, sleeping, eating, discomfort and symptoms, speech, physical appearance, school and hobbies, mobility, friends, mental function, excretion and depression). The results were compared with an age-matched reference population that included 163 boys from the Finnish capital-city area. The study protocol is registered to ClinicalTrials.gov (registration number: NCT01797718).

**Results:**

At baseline, the mean 16D score of the CDGP boys was similar to the age-matched reference population (0.95 vs 0.96, p=0.838). However, the physical appearance score (satisfaction with general appearance, height and weight) was significantly lower in the CDGP boys (0.75 vs 0.92, p=0.004) than their peers. Twelve months after treatment, Appearance had improved significantly (0.75 vs 0.87, p=0.004) and no HRQoL dimension was inferior compared to the age-matched reference population.

**Discussion:**

In terms of HRQoL, the main impact of delayed puberty was dissatisfaction with physical appearance. Puberty promoting therapy was associated with a positive change in perceived appearance, with no clear difference between low-dose testosterone and letrozole treatments.

## Introduction

Approximately 2-2.5% of healthy male adolescents experience delayed puberty, which is defined as the absence of pubertal maturation at an age of 2-2.5 SD later than the population mean (testicular enlargement in boys at age 14 years) (1). Constitutional delay of growth and puberty (CDGP) explains 63 to 83% of delayed puberty cases in boys (2, 3). Despite its self-limited nature, CDGP has been associated with diverse negative psychosocial effects, such as lower self-esteem, increased risk for depression, stress from social problems with peers and risk for substance use (4–7). In addition, pubertal delay may decrease participation in athletic activities, impair academic performance, and lead to unhealthy defense mechanisms and the development of unfavorable personality traits (4, 8–11). However, prospective studies in populations that screen for puberty disorders are scarce, and little is known about the actual effects of puberty-promoting treatment on health-related quality of life (HRQoL).

HRQoL is a multidimensional concept that includes both physical and emotional as well as social components associated with various diagnoses or specific treatments (12), but can also be used to assess healthy persons. To the best of our knowledge, no previous study has addressed the impact of puberty-promoting treatment on HRQoL among boys with CDGP. Herein, we investigated HRQoL in boys with CDGP who were randomized to receive testosterone or aromatase inhibitor letrozole treatment to promote pubertal development.

## Methods

HRQoL was investigated in 22 boys with CDGP, who participated in a randomized controlled trial in four Finnish pediatric endocrinology outpatient clinics between 2013 and 2017 (13). The boys were randomized to receive either aromatase inhibitor letrozole (2.5mg/day; n=11) or intramuscular testosterone (1mg/kg/every 4 weeks; n=11) for 6 months and followed up to 12 months since the commencement of treatment. The inclusion criteria, detailed study design, and results regarding study primary endpoints (changes in testicular volume, serum testosterone, luteinizing hormone (LH), follicle-stimulating hormone (FSH), inhibin B and urinary LH 6 months after initiation of treatment) have been reported previously (13). In brief, the boys were evaluated at 0-, 3-, 6-, and 12-months visits, their height, weight, and testicular volumes were measured, and pubertal stage was recorded.

In addition to a physical examination and blood tests, HRQoL was assessed with a generic 16D**^©^** HRQoL instrument, which is based on the respective adult version (15D^©^) but developed and validated for adolescents aged 12 to 15 years. Several studies have shown that the instrument is valid in the sense that it produces credible results and is capable of differentiating between healthy adolescents and patients in a wide range of health problems (14–19). The 16D includes 16 dimensions (vitality, sight, breathing, distress, hearing, sleeping, eating, discomfort and symptoms, speech, physical appearance, school and hobbies, mobility, friends, mental function, excretion and depression) with five response options for each. A set of population-based preference or utility weights is used to generate dimension level values and a single index score (16D score) on a 0 -1 scale (0 = being dead, 1= no problems), representing overall HRQoL (20). The minimum clinically important difference in the 16D score is 0.015 (21). The 16D questionnaires were included in the protocol after the first study visits of the first eight patients, and therefore 22 out of the 30 originally recruited boys were included in the analysis.

The study protocol was registered to ClinicalTrials.gov (registration number: NCT01797718) and approved by the Finnish National Committee on Medical Research Ethics and the Finnish Medicines Agency. Written informed consent was obtained from all subjects.

The data are presented using means and standard deviations (SD). Analyses were performed with SPSS for Windows (version 22.2, Chicago, IL). To compare changes in the 16D dimensions during the 12-month period, paired (within-group) and independent samples (between-group comparisons) t-tests were used. To account for the between-group difference in the baseline 16D score, the difference in the change of the 16D score from baseline to follow-up was estimated by using regression analysis with the baseline 16D score and treatment group dummy (0/1) as covariates. Correlations between calendar age, testicular volume, testosterone level, height (standard deviation score, SDS), the 16D dimensions and the total 16D score were analyzed with Spearman’s rho-test. The study population and the age-matched reference population were compared using independent samples T-test. The age-matched reference population included 163 boys from the Finnish capital-city area and was collected in 2013 (22). All p-values were two-tailed, and the statistically significant level was set to p-value less than 0.05.

## Results

**Figure 1:**
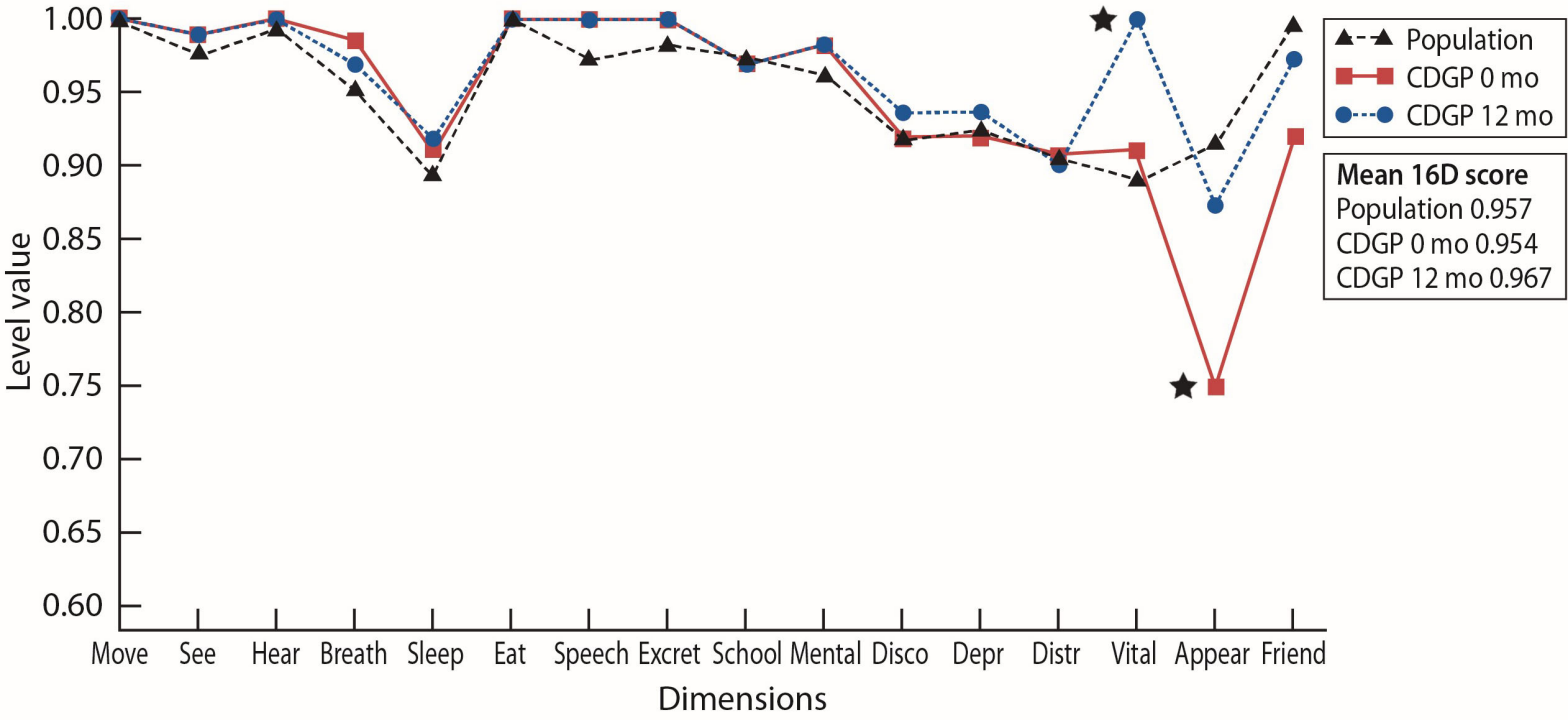
The mean 16D profiles in male patients with CDGP (n=22) compared with age-standardized general male population (n=163). A higher score indicates a better HRQoL. The star-symbol indicates a statistically significant (p<0.05) difference between age-standardized general male population and the CDGP male patients.

Pubertal stage, testicular volume, and testosterone serum concentrations at baseline and at 12 months, *i.e.*, 6 months after the cessation of puberty-promoting treatment, are shown in **Tables 1a, b**. At baseline, the mean 16D score of the CDGP boys was similar to the age-matched reference population (0.95 vs 0.96, p=0.838). However, the physical appearance score (indicating satisfaction with general appearance, height and weight) was significantly lower in the CDGP boys than in controls (0.75 vs 0.92, p=0.004) (**Figure 1**). As the pubertal maturation level at baseline varied very little among the subjects, with all boys showing only the very first signs of pubertal activation (**Table 1a**), we also used age at baseline as a marker of pubertal delay. Older age at baseline showed a significant correlation with the Friends-dimension (r= -0.53, p=0.012) but with no other HRQoL dimension score (r= -0.39 to -0.09, p=0.076 - 0.677). Baseline height SDS, serum testosterone level or testis volume showed no significant correlations with the HRQoL scores (r= -0.40 to 0.16, p=0.069 - 0.968).

Table 1Characteristics of the study population at baseline (a) and at 12 months (b), *i.e.*, six months after cessation of treatments.

**Table:** 

Table 1a: Baseline characteristics (mean and range)		
	Letrozole (n=11)	Testosterone (n=11)
Age (years)	14.7 (14.2 - 15.3)	15.1 (14.1 - 16.2)
Testicular volume (mL)	2.9 (1.2 - 4.8)	3.6 (2.3 - 5.0)
Height (cm)	153.1 (141.9 -165.4)	158.3 (149.0 - 168.3)
Height (SDS)	-2.3 (-3.6 - [-0.5])	-1.7 (-2.7 - [-0.5])
Tanner G-stage distribution (n, stages 1-5)	4/7/0/0/0	2/9/0/0/0
Tanner P-stage distribution (n, stages 1-5)	7/4/0/0/0	3/8/0/0/0
BMI (kg/m2) at 0 months	20.8 (16.0 - 31.1)	20.0 (16.2 - 28.8)
Testosterone (nmol/L)	1.8 (0.7 - 4.5)	2.8 (1.5 - 4.5)

BMI, body-mass index; SDS, standard deviation score.

**Table:** 

Table 1b: Follow-up data at 12 months (mean and range)		
	Letrozole (n=11)	Testosterone (n=11)
Age (years)	15.7 (15.2 - 16.3)	16.0 (15.0 - 17.2)
Testicular volume (mL)	12.6 (4.9 - 18.3)	9.4 (5.7 - 12.9)
Height (cm)	161.7 (150.1 - 173.2)	165.7 (156.7 - 176.4)
Height (SDS)	-2.1 (-3.8 – [-0.1])	-1.5 (-2.8 - 0.0)
Tanner G-stage distribution (n, stages 1-5)	0/1/4/6/0	0/0/4/7/0
Tanner P-stage distribution (n, stages 1-5)	1/2/5/3/0	0/0/4/7/0
BMI (kg/m2) at 12 months	21.0 (16.4 - 28.2)	20.7 (16.6 - 28.7)
Testosterone (nmol/L)	10.0 (5.5 - 18.3)	12.1 (6.5 - 18.0)

BMI, body-mass index; SDS, standard deviation score.

At baseline, the testosterone-treated group showed a lower mean 16D score (0.93 vs 0.98, respectively, p=0.044) and lower scores on the dimensions of depression (0.84 vs 1.00, p=0.044) and friends (0.84 vs 1.00, p=0.044) than the letrozole-treated subjects, respectively.

After the 12 months of follow-up, the physical appearance-dimension improved significantly (0.75 vs 0.87, p=0.004). When assessing the two treatment groups separately, the mean 16D score improved in a clinically important and statistically significant manner in the testosterone group (0.93 vs 0.96, p=0.019). Also, the score of the physical appearance dimension in the testosterone group improved significantly (0.70 vs 0.84, p=0.018), whereas in the letrozole group no significant changes in the mean 16D score or individual dimensions were found (16D score 0.98 vs 0.98, p=0.837, physical appearance 0.80 vs 0.92, p=0.104). However, neither the change in the mean 16D score nor on the individual dimensions differed significantly between the treatment groups (p=0.056 – 0.814). The change in testicular volume or testosterone level during follow-up showed no correlation with the change in physical appearance or the total 16D scores (p=0.412 - 0.872). At the 12-month visit, however, no significant differences in HRQoL or its dimensions were found between the treatment groups (p=0.167 – 1.000). Further, neither the 16D score nor any of its dimensions were impaired compared to the age-matched reference population; in fact, the boys with CDGP had higher vitality scores (**Figure 1**).

## Discussion

We found that in terms of HRQoL, self-limited delayed puberty mainly affected dissatisfaction with physical appearance, with no clear negative impact on the overall well-being of the adolescents as measured by the 16D. Puberty promoting therapy was associated with positive changes in dimensions of physical appearance and vitality, and we found no clear differences in the effects of low-dose testosterone and letrozole treatments on HRQoL. Pharmacological treatment of delayed puberty in male adolescents with either testosterone or letrozole is effective in promoting the physical signs of puberty (13). However, to the best of our knowledge no previous studies have assessed the impact of puberty promoting treatments on HRQoL in CDGP males, although the psychosocial burden related to delayed puberty is the main indication for medical treatment.

Our results suggest that males with CDGP experience body dissatisfaction prior to treatment. Such discomfort and feeling different from peers at this vulnerable stage of life could complicate psychosocial development and interaction with peers (4, 5, 7, 23, 24). In a report from the UK, body image was the third most important concern among adolescents after lack of employment opportunities and educational failure. Some males were ready to consider taking steroids to achieve their appearance goals and nearly a quarter of males avoided participation in physical education due to worries regarding their physical appearance (25). Boys may also be more dissatisfied with their lack of muscles than their short stature (26, 27).

Although the dimensions of depression and distress showed no impairment in comparison with the age-matched general population, older age at baseline age correlated negatively with the dimension friends suggesting that more severely delayed puberty limits participation in social activities. This view is supported by our previous findings in male patients with congenital hypogonadotrophic hypogonadism (CHH), a more severe form of pubertal delay, where the overall HRQoL (measured by the 15D) and dimensions of depression and distress were lower than among the general male population. In the CHH population, age at diagnosis correlated inversely with overall HRQoL indicating that a delayed start of androgen replacement therapy may have a lasting adverse influence on HRQoL (28).

In this study, the mean 16D score remained unchanged whereas the HRQoL dimensions of physical appearance and vitality improved significantly after puberty promoting treatment. Puberty promoting treatment thus seems beneficial for body image, although the lack of an untreated control group must be acknowledged.

HRQoL has been studied in some pediatric populations with delayed puberty related to chronic disease during the past few years. For example, Menon et al. found no correlation between delayed puberty and HRQoL (measured by the Pediatric quality of life inventory, PedsQL, and Cardiac Module) in young Fontan survivors (29), whereas Wood et al. showed that testosterone therapy for pubertal induction was associated with an improvement in HRQoL (measured by the PedsQL) in males suffering from Duchenne muscular dystrophy (30). Further, in males, delayed puberty has correlated negatively with both ego development and sexuality (9) and testosterone therapy has been shown to have a positive effect on self-perceived job competence and athletic abilities (31). Our findings are in line with Rohyaem et al. (32), who found that boys with hypogonatropic hypogonadism (HH) had pervasive and persistent concerns about body image and concluded that in boys with HH gonadotropin treatment clearly enhanced HRQoL (measured by Inventory for assessment of quality of life in children and adolescents, ILK).

The main strength of the current study is the well characterized study population in terms of physical and hormonal markers of puberty (13). Further, the 16D questionnaire is well validated in the Finnish population and has a wide range of dimensions *a priori* relevant for measuring the effects of CDGP. Limitations of the current study include the limited number of participants, resulting in modest power to detect subtle differences in HRQoL. Our research was originally powered for primary endpoints (changes in testicular volume and hormonal markers of puberty) and QoL results should be considered exploratory. Further, our results reflect findings in boys who consider medical treatment of CDGP necessary and may not be generalizable to all boys with CDGP. Although the 16D questionnaire functions well in both healthy and unhealthy adolescents it is a generic questionnaire and not designed specifically for delayed puberty. Given our results, we suggest the additional use of questionnaires that specifically address body image and social functioning in future studies of boys with CDGP.

In conclusion, our findings in boys with CDGP combined with previous findings in cohorts of males with more severely delayed puberty (CHH) suggest that the delay in pubertal progression first affects body satisfaction and in more severe delay also general psychosocial well-being. As a clinical consequence, school health care and outpatient clinics that serve adolescents should evaluate pubertal status and recognize adolescents presenting with apparent delay in pubertal development, as dissatisfaction with the condition may be ameliorated with puberty-promoting treatment.

## Data availability statement

The datasets presented in this article are not readily available because of the Finnish legislation on individual data sharing. Anonymised data can be shared upon reasonable request to the corresponding author, taking account Finnish legislation on individual data sharing. Requests to access the datasets should be directed to MH, matti.hero@hus.fi.

## Ethics statement

The studies involving human participants were reviewed and approved by The Finnish National Committee on Medical Research Ethics and the Finnish Medicines Agency. Written informed consent to participate in this study was provided by the participants’ legal guardian/next of kin.

## Author contributions

LK has contributed in gathering and analysing the data, making the figure and tables and in writing the article. TV has contributed by designing and completing the original study, gathering and analysing the data and in manuscript preparation. HH, ST, RV and JT were all active during the actual clinical study and contributed in the study design, reqruiting and examining patients and gathering data as well as manuscript preparation. SK contributed to data analysis and writing the manuscript. HS contributed in the original study design and analysing the data, developed the original 16D questionnaire and writing the manuscript. PM, MH and TR have been active in the entire research process from study design, patient recruitment and examination to data analysis and commenting the manuscript. All authors contributed to the article and approved the submitted version.

## Funding

This study was funded by the Finnish Medical Foundation, the Foundation for Pediatric Research (grant number 160262) and Helsinki University Hospital Research Funds. Each of these founder’s have given a personal grant for LK for conducting PhD research.

## Conflict of interest

HS is one of the developers of the 16D.

The remaining authors declare that the research was conducted in the absence of any commercial or financial relationships that could be constructed as a potential conflict of interest.

## Publisher’s note

All claims expressed in this article are solely those of the authors and do not necessarily represent those of their affiliated organizations, or those of the publisher, the editors and the reviewers. Any product that may be evaluated in this article, or claim that may be made by its manufacturer, is not guaranteed or endorsed by the publisher.
